# Comparing Human Factors for Augmented Reality Supported Single-User and Collaborative Repair Operations of Industrial Robots

**DOI:** 10.3389/frobt.2019.00037

**Published:** 2019-05-31

**Authors:** Doris Aschenbrenner, Florian Leutert, Argun Çençen, Jouke Verlinden, Klaus Schilling, Marc Latoschik, Stephan Lukosch

**Affiliations:** ^1^Faculty of Industrial Design Engineering, Delft University of Technology, Delft, Netherlands; ^2^Mobile Systems, Zentrum für Telematik, Würzburg, Germany; ^3^Faculty of Design Sciences, University of Antwerp, Antwerp, Belgium; ^4^HCI Group, Institute of Computer Science, Julius-Maximilians-Universität, Würzburg, Germany; ^5^Faculty of Technology, Policy and Management, Delft University of Technology, Delft, Netherlands

**Keywords:** augmented reality, mixed reality, maintenance, repair, industrial robot, user study, collaboration, collaborative mixed-reality application

## Abstract

In order to support the decision-making process of industry on how to implement Augmented Reality (AR) in production, this article wants to provide guidance through a set of comparative user studies. The results are obtained from the feedback of 160 participants who performed the same repair task on a switch cabinet of an industrial robot. The studies compare several AR instruction applications on different display devices (head-mounted display, handheld tablet PC and projection-based spatial AR) with baseline conditions (paper instructions and phone support), both in a single-user and a collaborative setting. Next to insights on the performance of the individual device types for the single mode operation, the study is able to show significant indications on AR techniques are being especially helpful in a collaborative setting.

## 1. Introduction

As digitization advances further, there is the need to point production industry in the right direction when it comes to using Augmented and Mixed Reality (AR and MR) in their everyday applications. The ongoing Industry 4.0 or “smart factory” discussion initiated a transformation process within the automation industry which encouraged industrial decision makers to think out of the box in a couple of fields, including human-machine-interfaces. If we consider trends like human-robot co-production, reconfigurable production plants and one-off production, it is clear that these also lead to new challenges for the human operators of those systems. Furthermore, an increasing amount of complex work tasks can be expected due to this transformation, which requires more skilled and experienced workers. This is especially true for the field of maintenance and repair, where more complexity also implies more possible error sources. Globalized service requests are ever increasing today, leading to a distinct need for better support mechanisms for knowledge transfer and support.

Augmented Reality has been used for maintenance applications since the field started to evolve, because it allows context-aware visual guidance for task execution. This could be profitable for both local and remote maintenance scenarios: a local worker could benefit from pre-stored instructions, while an external expert can support a local worker with remote instructions. Past research was able to effectively demonstrate the possibilities and benefits of AR in those contexts, which will be outlined in the related work section. As AR finally seems to be ready for the mass market, we are encountering a hype-driven momentum to implement it into real world applications as effective “smart factory” demonstrators.

This increasing demand leads to very practical questions for each new AR application development: Which device and which visualization and interaction methods should be used? At the beginning of this work, our team has been motivated by an industrial partner during a research project to answer this “simple” question for a given repair task. From human-computer interaction theory, we know very well that this answer strongly depends on the specific application, the implementation of the AR application, and the user group: A good interface always needs to be optimized for the specific setting. Nevertheless, within the field of two-dimensional screen interfaces, there have been several successful attempts for guidelines or general design principles which are true for more than one specific case. Why is it so hard to give at least some general answers or inquiry directions to the above question?

One of the main problems is comparability from a scientific perspective. From a practical point of view, the question whether to use a tablet PC or a head-mounted display is highly relevant. From a theoretical point of view, it can be argued that those devices imply highly different interaction methods, and thus are not comparable at all. The same is true for the practical question whether audio or text instruction should be used in the application, or questions regarding the preferred type of visual cues for a range of different devices. On top of this incomparability, we cannot deduce the performance of a specific interaction paradigm in general, as user tests will always test the device capabilities and not the paradigm directly. It is well known that for example a poor tracking algorithm performance due to latency or camera resolution can influence the usability of an application in such a way, that a comparison of the interaction paradigm itself is not possible. But is the solution really to refrain from quantitative tests in the area of those questions? One could argue as well, that comparing a new AR application to a paper instruction baseline suffers from the same shortcomings although it is the common approach for a new method. In our eyes, the practical demands to “compare apples and oranges” cannot be neglected, which is why our team started to explore the questions from an output-oriented real world application point-of-view, fully aware of the limitations of the generalizability of the acquired results.

Our aim is to look into possible support for single-user and collaborative repair cases with the help of specific Augmented Reality or Mixed Reality applications on different devices. Whereas AR/MR research has a long history of trying to assist a single user with repair tasks using an automated system, collaborative mixed-reality applications (CMR) promise to provide a computer supported collaborative work environment where another user (geographically remote but virtually co-located) assists the local worker. Of course, the overall team performance is influenced by both interfaces, the one used by the expert and the other used by the remote worker. Our current attempt is to keep one of the sides fixed in order to investigate the other side. Our previous work focused on situation awareness of the expert comparing different MR implementations (Aschenbrenner et al., 2017 and Aschenbrenner et al., 2018a), this research investigates the influence of different AR implementations on the performance and the individual perception of the local worker.

Next to investigating which specific AR/MR implementation leads to a better performance for a specific task, it is important to compare a collaborative use case with a single-user use case in order to draw more general conclusions: How does a collaborative setting influence the measured human factors of the same task compared to single-mode operation? To which extent can we apply our knowledge on single-user repair support? Can we draw conclusions on the performance with AR support in general?

This article compares the results of three user studies exploring different AR methods with single-user and collaborative use cases. For all studies, we investigate the same realistic repair case: the exchange of a malfunctioning servo amplifier in an authentic switch cabinet of an industrial manipulator. This task is a common repair case used for failure detection and its correct conduction is required for the industrial robot to function. We identified this specific task during a contextual inquiry on the field of remote maintenance for industrial robots (Aschenbrenner et al., 2015).

The aim of this paper is to publish the studies in a comprehensible overview. Although part of the work has already been published, the three studies have been carried out on the same task, which allows at least some comparability with respect to the performance of different AR devices.

This article first analyses and discusses related work in the next section, before explaining the experiment's scenario, procedure and data acquisition in detail. The results of the individual studies and the comparison of all studies are presented. The last section discusses the findings.

## 2. Related Work

Augmented Reality (AR) is becoming more ubiquitous in the everyday world as more and more consumer products become available, for example AR applications on smartphones or devices like the Microsoft Hololens or the Magic Leap. AR is a human computer interaction technique to augment the natural human perception with additional information (Azuma, 1997). In contrast to an all-virtual immersion in Virtual Reality, AR is found on a Mixed Reality (MR) continuum between reality and virtuality as introduced by Milgram and Kishino (1994).

Still, there are several benefits of AR applications mentioned by Anett Mehler-Bicher (2011) that also apply for MR:

1. Computer supported enhancement of human senses with additional digital objects.
2. The possibility to visualize detailed and complex information.
3. The ability to support complex and difficult tasks.
4. Minimization of time-to-content (the time needed for getting the required content).
5. Possible combination of haptic and digital user experience.

But those benefits are not achieved automatically. There is a long history regarding AR assistance on repair, maintenance or manual assembly. Although the latter is different from the repair operations targeted in this publication they can be compared. In both, a specific task needs to be solved which follow the same structure: part identification, handling, alignment, joining, adjustment, and inspection (Nof et al., 2012).

Early research on AR for production tasks has been performed with test persons who assembled a construct of Duplo blocks (Tang et al., 2002), showing that the probands with AR equipment made less errors. There has been no difference regarding the amount of time used. In such study designs, typically one group used a paper-instruction as a baseline, the others use the various AR applications, in this case an LCD display and a head-mounted display (HMD). Earlier research showed that complex tasks with a high search effort can be supported with AR (Reinhart and Patron, 2003). With such support, users prefer visual notifications over audio notifications on recent changes in the AR environment (Cidota et al., 2016a,b). Performing maintenance or repair actions can be faster, more productive and more flexible due to the direct spatial context of the information.

Recently, Dey et al. (2018) published a comprehensive survey of influential AR papers with user studies which have been published between 2005 and 2015. The study found 30 papers for the area “industrial". Compliant with Kim et al. (2018), who analyze ISMAR papers from 2008 to 2017, they come to the conclusion, that systematic user studies have increased within the AR domain, but that there is the definitive need for more publications and user studies on collaborative systems.

The first studies in the area of collaborative work (Flor, 1998) investigated the conversations and information exchange of two workers working side by side on an assembly task. Those conversations were mainly the identification of goal objects, the instruction for tasks and the confirmation of finished tasks. Further observation studies (Tang, 1991) introduced a common work field, which can be used for sketches and writings. It has been shown that this common view helped to support the work process and was able to convey information better. For a remote constellation, research by Kuzuoka (1992) showed, that collaborative groups on the same workplace performed better than spatially separated coworkers. It also showed the potential of multimedia systems: without the shared context provided by an additional application, the spatially separated workers seem to show a poorer performance.

Another study tried to determine which visual information provides a benefit for a coworking team. The study by Kraut et al. (2003) compared the performance of a person working alone on a bicycle repair task with the performance of a group of a remote mechanic and a local worker, who were working on the same task. The experiment provided evidence for the importance of a shared visual context in remote collaborative work. Further investigations by Gergle et al. (2013) on puzzle tasks showed, that the shared visual context was also important for situation awareness and grounding.

Regarding specific applications of AR within collaborative settings, research showed that AR can be of use in collaborative scenarios by establishing a virtual co-location (Datcu et al., 2014). Such virtual co-location can provide collaborating users with a shared visual context and improve the team situational awareness (Lukosch et al., 2015b). Still, further research is necessary to identify how remote users can interact with the local users and how their presence and awareness can be improved (Lukosch et al., 2015a).

A lot of studies on AR used a neutral application domain by relying for example on Lego blocks or puzzles. In contrast to those, this article focuses on an application-oriented industrial context. Table 1 gives an overview of recent research and user studies on the application of AR for a specific industrial application.

**Table 1:** Overview industrial maintenance with AR.

**Publication**	**s/c**	**Compared conditions**	***N***	**Result**
Chen et al. (2013)	c	Tablet tracking vs. tablet video	16	ns
Fiorentino et al. (2014)	c	Paper vs. large screen	14	Large screen sign. better
Gauglitz et al. (2014)	c	Tablet tracking vs. tablet video	60	Tracking sign. better
Gonzalez-Franco et al. (2016)	c	Face to face vs. HMD	24	ns
Henderson and Feiner (2011)	s	HMD vs. screen	13	HMD sign. better
Marner et al. (2013)	s	SAR vs. screen	24	SAR sign. better
Radkowski et al. (2015)	s	Paper vs. screen	33	ns
Rosenthal et al. (2010)	s	SAR vs. screen	30	SAR sign. better
Webel et al. (2013)	s	Instruction video vs. AR	20	ns /errors AR sign. better
Zhu et al. (2013)	c	Paper, video, interactive	8	ns

*s, single; c, collaborative; N, amount of participants; ns, not significant; sign., significant*.

What can we learn from this overview? There is a strong indication that AR can be helpful in a repair scenario—both in single and in collaborative mode. The results indicate that there are three main classes of devices: HMDs, tablet PCs/handheld devices and projection-based spatial Augmented Reality (SAR). But unfortunately, they are not able to give an answer to the above mentioned practical question. First of all, a lot of studies are not able to confirm the laboratory results of a better AR performance for the real application, as those tend to be more complex and require specific domain knowledge. Second, most of the user studies compare their new implementation with a baseline condition and have been able to show a significant superiority of the specific AR implementation regarding task duration, error, task load and individual rating. But there are very few comparisons between different devices. And third, a lot of participants come from the easy accessible group of students and not from the target user group. Thus it can be concluded that there is clearly a lack of research regarding collaborative scenarios, comparisons between different device implementations and comparison between single-mode and collaborative setting.

## 3. Experiment Description

**Figure 1:**
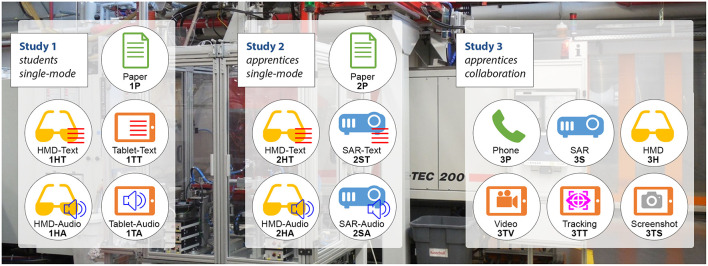
Overview of the three studies: For the same repair task, each icon represents a specific device configuration. Each condition is identified with the study number, device identifier and variation identifier and for each, the repair task was performed by 10 participants.

A visual overview of the studies presented in this paper is provided in Figure 1. There are sixteen different test groups, while each consists of 10 participants. In order to provide a better overview, a naming system consisting of three letters is introduced. The first letter is the study number (1, 2, or 3). The second letter represents the different devices: head-mounted display (H), tablet PC (T) and spatial Augmented Reality or projection-based AR (S). The letter “P” is used for the baseline conditions: paper instructions in the first and second experiment, and phone in the third experiment. The third letter indicates the specific variation, if any have been tested within the same device. In study 1 and 2, this is text (T) or audio (A) for all AR implementations, and in the third study, there is the differentiation of video (V), tracking (T) or screenshot (S) for the tablet PC conditions. Furthermore, each group has a different icon as shown in Figure 1, while the different devices are highlighted by the main icon and the color.

The first study was performed with 50 student participants and compares five different groups: an AR implementation for a head-mounted display (HMD) with either audio or text instructions, a tablet PC based AR application with either audio or text instructions and a baseline of paper instructions.

The second study involves 50 participants (technician apprentices) in a non-collaborative setting and compared the following five groups: HMD with either text or audio, projection-based spatial AR-implementation (SAR) with either text or audio and the baseline of using paper instructions. We chose to collaborate with a technician school for apprentices trained in similar maintenance tasks, because the students of the first study clearly lacked domain knowledge.

The third study was performed in collaboration with the same school with 60 different participants in six different groups in order to investigate the collaborative setting. The same researcher performed the role of an external expert during the study, helping the participants with the task. He used the same paper instructions that were used during study 1 and 2. The study compared a HMD application, three different tablet PC applications (video without annotations, visual annotations on a screenshot and tracked visual annotations) and a projection-based SAR application to a baseline of support via phone.

All studies followed the same instruction set provided by the robot manufacturer as explained in section 3.1. In order to omit learning effects, all user studies relied on different participants (between subject design), resulting in 160 participants in the end. The requested repair operation took on average half an hour. Together with questions and questionnaires each iteration took approximately 1 h. The precise procedure is described in section 3.2. In section 3.3, we specify the objective and subjective measurement methods. Finally, we present the overview of the used media devices in section 3.4.

### 3.1. Repair Scenario

In general, failures that reduce a plant's availability occur unexpectedly and require a fast intervention by the machine operator or service technician, in order to avoid an imminent production breakdown in the worst case if the failure occurs in a critical production path. The failure cause can be multifaceted. However, using their experience, service technicians can often find the error cause and correct the faults. As the interviews with employees of industrial partners indicated, hardware malfunctions inside the robot are extremely hard to find, because the cause is not directly visible. Especially if the malfunction is located inside the switch cabinet, an effective support for failure localization and removal is needed.

The contextual analysis of past projects on telemaintenance on industrial robots (Aschenbrenner et al., 2015) showed, that the exchange of a malfunctioning servo amplifier (also called “controller”) with a new one is performed as a standard means of failure handling. There are some error messages of the robot indicating a “controller error.” The technician then tries to exchange the controller. If the error is gone after the repair operation, the plant is ready to restart. If the error is still present, it has occurred due to a different reason, often broken cables.

**Figure 2:**
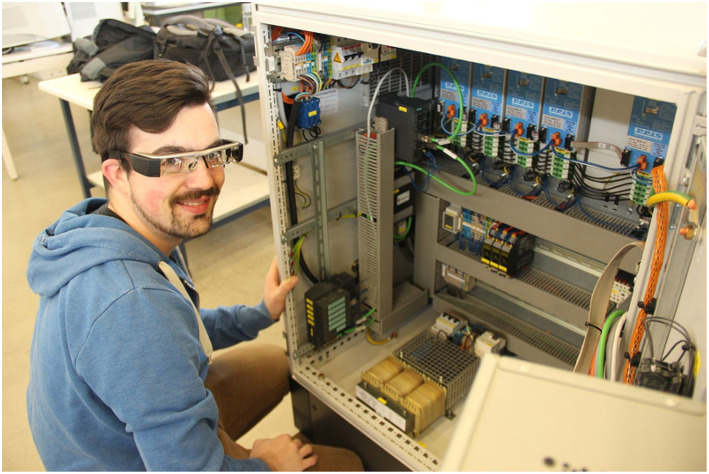
The switch cabinet with integrated servo amplifies (blue).

Each servo amplifier located in the switch cabinet (blue boxes in Figure 2) controls one robot axis. They are interconnected with different cables and plugs and can only be exchanged in a complicated extraction procedure.

Connecting the plugs in the right order is mandatory for installing or dismounting the controller. If a cable is connected the wrong way, short-circuits may occur inside the plant or the robot may drive erroneously. This may cause a complete breakdown of the plant, the destruction of expensive robot parts or in the worst case injury to people. In order to protect property and personnel, it is indispensable to omit failures and increase the diligence of the maintenance operation.

In the specific case of a controller exchange in the switch cabinet, apprentices or new employees need extensive training in order to enable them to perform this maintenance action on their own. As already a small mistake may lead to severe consequences, their work needs to be supervised by a trained employee, or at the very least, the result needs to be double-checked. Supporting an untrained worker by remote collaboration would help to minimize failures and obviate the need for review in the best case.

### 3.2. Experiment Procedure

Every participant undertook the mechanical task of removing the malfunctioning controller and inserting a new one with the help of a specific application. All participants signed an informed consent form which also grants the right to take and use pictures during task execution. All studies have been carried out with a between-subject study design in order to omit learning effects: if a participant has conducted the repair operation once with for example the handheld PC, it will be easier for him or her to perform it a second time with a different support application, which makes a comparison of the performance of both devices complicated or impossible. As it surely would be very interesting to measure, if and how AR can support learning for those kind of tasks, a different study would be necessary. Normally, we would randomize the application, which the participant will use on the spot (in order to omit other influences). In this user study, this was not possible, because the setup process for each AR application takes too long. We therefore chose specific days for specific applications and tried to randomize the participants. In the first study, the students were recruited from the HCI proband system of University of Würzburg. Experiments were conducted in a dedicated and undisturbed environment. For the second and third study, the technician school Franz-Oberthür-Schule provided a separate room. Although the specific room was different, the setup of the switch cabinet was exactly the same. As the worker is really surrounded by the cabinet during task execution, we think that the change of the rest of the environment does not cause too much differences, as long as is it equally quiet. Nevertheless, it would be interesting to carry out such a study at the real production site, because the noise level and other disturbances are much higher there (for example automated guided vehicle systems tend to pass the worker during task execution), but is very hard to keep those distractions consistent during the entire study.

Each experiment had the following structure:

1. Pre-exercise questionnaires
2. Introduction of task and AR application
3. Exploration phase of specific AR application
4. Exercise: controller exchange
5. Post-exercise questionnaires
6. Qualitative feedback

At the time of the study, ethical approval was not required in line with institutional guidelines and national legislation. As the university of Würzburg did not have an ethic review board at the time the studies started, the study was performed with regards to the standard of user tests in mind (Shneiderman, 1992; Nielsen, 1993; Dumas et al., 1999) and there are nearly no known risks of using AR (recently confirmed by Vovk et al., 2018).

### 3.3. Collected Data

The study focuses on quantitative data, but allowed additional qualitative feedback. We collected both objective (time measurement of task duration, measurement of amount of errors) and subjective data with standardized and validated questionnaires. In the first study, we used the questionnaire QUESI (Anja and Jörn, 2010) to assess usability and the NASA-TLX (Hart and Staveland, 1988) questionnaire to assess task load. The second and third study additionally used SART (Taylor, 1990) to measure situational awareness and ISONORM 9241/10 (Prümper, 1997) to access usability. The concept of situational awareness has been used in several other domains such as energy distribution, nuclear power plant operational maintenance, process control, maritime or tele-operations (Salmon et al., 2008). We used SART as this avoids the freezing of action during the test, compared to applying the SAGAT method (Endsley et al., 1998), as SART is administered post-trial and has a non-intrusive character. Furthermore, a post-test self-rating technique is applicable whenever “SA content is not predefined and the task is dynamic, collaborative, and changeable and the outcome is not known (e.g., real world tasks)” (Salmon et al., 2009). Finally, since the third study was the only study in a collaborative setting, we undertook an analysis of grounding effects.

After the repair operation, it was assessed whether the participant made any errors. The error description was obtained by a domain expert, who is responsible for training apprentices for this specific task. The following errors were counted:

- A cable is not plugged in its right place.
- A cable is not mounted correctly and can be loosened by hand.
- The plugs are not in the right place.
- The screws on the plugs are not tightened.

### 3.4. Used Media

#### 3.4.1. Paper Instructions: 1P and 2P

The instructions for the controller exchange have been provided by KUKA Industries and describe the different necessary working steps. In the context analysis of the entire project described in Aschenbrenner et al. (2015), the procedure has been documented by video and text. There are some necessary preparations like driving the robot to a reference position or switching off the voltage which were omitted in the used instructions, because we used a switch cabinet without any voltage or connected manipulator du to safety.

#### 3.4.2. Head Mounted Display: 1HA, 1HT, 2HA, 2HT, 3H

**Figure 3:**
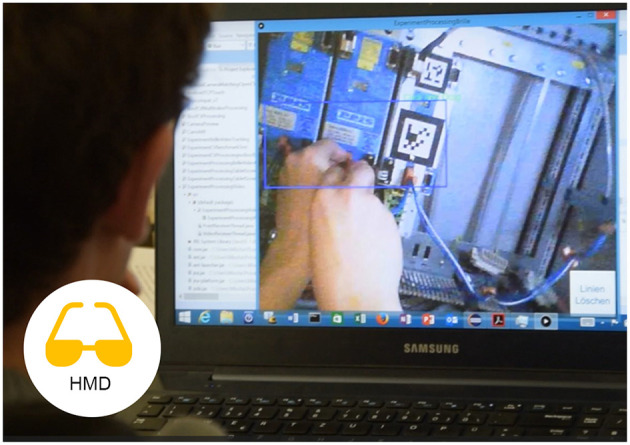
The external expert (role performed by a researcher) looking at the HMD transmission.

**Figure 4:**
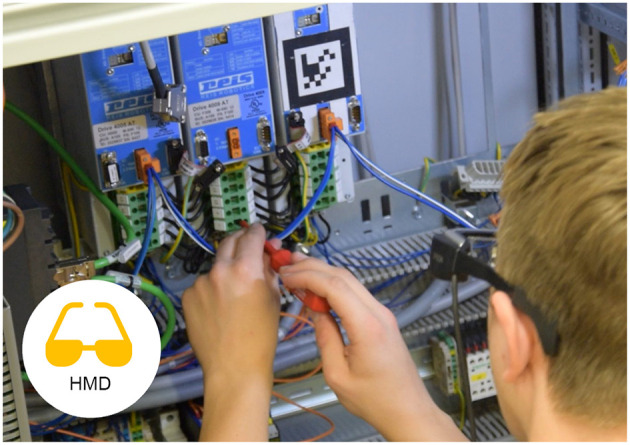
A participant working with the Head-mounted Display (HMD) following instructions.

A head-mounted display (HMD) is a display device worn on the head or as part of a helmet that has a small display optic in front of one (monocular HMD) or each eye (binocular HMD). An example can be seen in Figures 3, 4. For all experiments we used the Epson Moverio BT200. Those glasses have a LCD polysilicium display integrated for each eye, illuminated by mini projectors on the rim. The displays provide a resolution of 960 × 540 pixels and cover 23 percent of the field of view. There is an external control device with a touchpad, using a 1.2GHz dual-core-processor and 1GB RAM.

For the single mode, a Metaio SDK based implementation was used both with audio instructions and with text instructions. For the collaborative mode, an Android application has been implemented which connects to the desktop PC application of the remote expert via Wi-Fi and tries to visually track camera picture features at the location of the expert's annotations. Rojkov et al. (2017) contains the details of the tracking algorithms.

#### 3.4.3. Projection Based SAR: 2SA, 2ST, 3S

**Figure 5:**
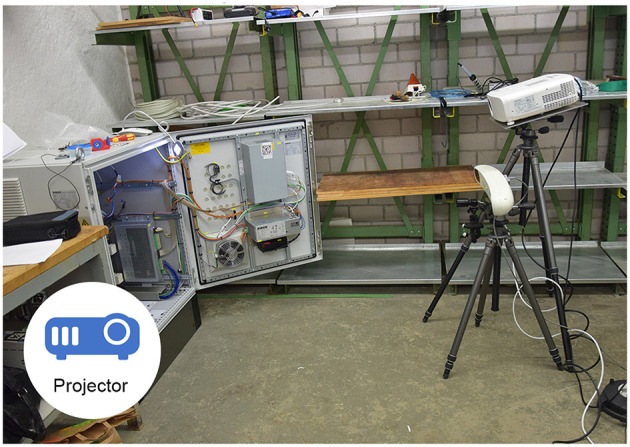
The setup for the projection based SAR application.

**Figure 6:**
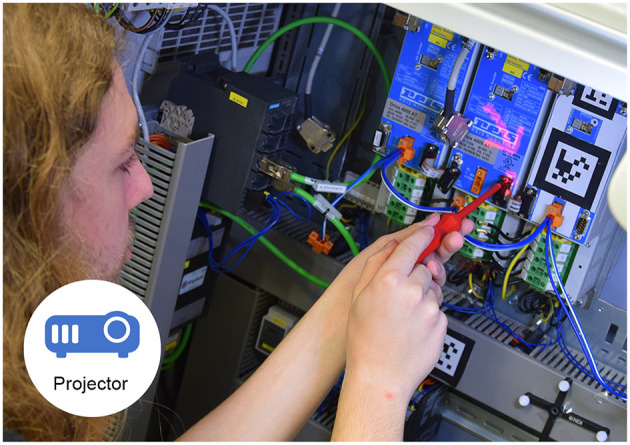
A participant working with the projection based AR application (pink annotation, arrow).

The projection based Augmented Reality application [or spatial Augmented Reality application as explained in Oliver Bimber (2005)] uses a Panasonic PT-VZ575N projector with a resolution of 1,920 × 1,200 pixels. Mounted rigidly to the projector is a PointGrey Blackfly camera, that offers a static third person perspective of the work surface. The setup can be seen in Figure 5 and the working participant in Figure 6. After the camera and the projector have been calibrated, a Structured Light approach is used to gather a surface model of the working site. With the help of this model, pre-configured visual cues can be projected for the single mode. In the collaborative mode, the expert can directly draw or send visual instructions to the surface of the working environment. The worker also can use a pointing device tracked by a Polaris Spectra system in order to annotate his view. More publications to this specific setup can be found at Leutert et al. (2013).

#### 3.4.4. Tablet PC: 1TA, 1TT, 3TV, 3TT, 3TS

Tablet or hand-held PCs have been commercially available for several years and are regularly used in everyday life as well as in industrial settings. This is why this device serves as a representative of the current common practice in industry. For a proof of concept in live production see Aschenbrenner et al. (2016), where a tablet computers for collaborative work in an active production environment.

For the single-mode, we used the Samsung Galaxy Tab2 with Android 4.0.3, screen size 10.1 inch, resolution 1,280 × 800 pixels, 3.2 MP camera with a Metaio SDK application with audio or text.

In the collaborative mode we used a ASUS MEMO ME302C Tablet with an 1.6 GHz Intel Atom Z2560 Processor with 2 GB RAM. The device runs Android 4.3 and the display has a resolution of 1,920 × 1,200 pixels. For the collaborative scenario, we compared three different applications: a version which provides just the camera picture, a version which enables the expert to make a screenshot and annotate it, and a permanent tracking approach, which is described in Rojkov et al. (2017).

**Figure 7:**
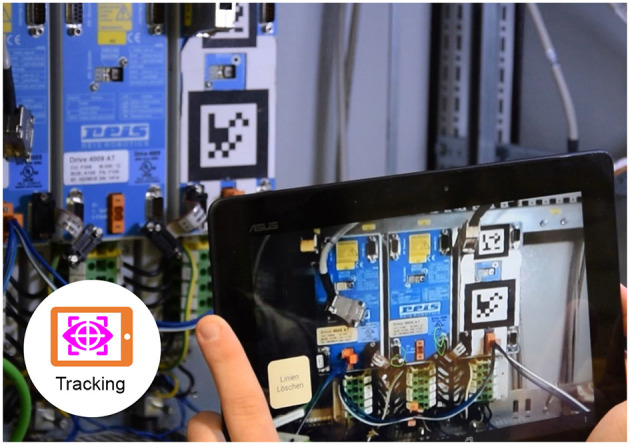
A participant working with the tablet PC AR application with tracked annotations (green).

**Figure 8:**
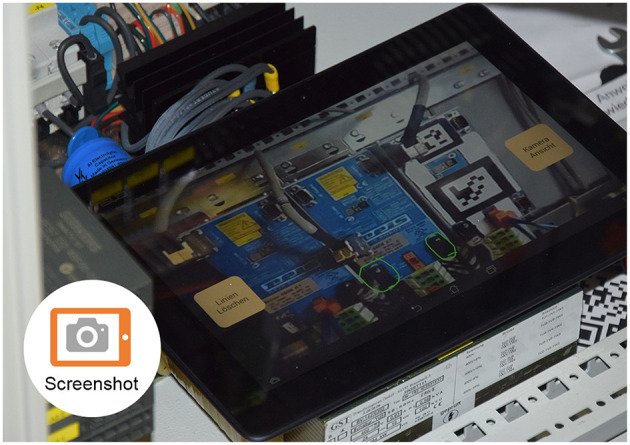
The screenshot tablet application (participant needed to lay down tablet PC).

An example of the implemented video-based implementation which uses feature-based tracking can be seen in Figure 7. The screenshot-based alternative can be seen in Figure 8.

#### 3.4.5. Phone Condition: 3P

In the collaborative experiment, we chose contact between worker and expert without shared visual context by introducing a “telephone” condition. This condition was meant to provide a baseline to the AR application experiments. In our experimental setup for the collaborative case, both users cannot see each other but can have a normal conversation in the same room. This is the optimal condition which eliminates all latency of the audio, and this audio condition is used for all of the experiments. There is of course an influence of the audio quality and latency on the quality of computer supported collaborative work, but it is intentionally left out in this experiment in order to be able to compare only the visual conditions.

## 4. Results

This section summarizes the results of the studies from a statistical point of view. In order to get an overview of the measurements, the next section starts with boxplots displaying a specific factor measured in all experiments. In the following section, we only compare the different media applications with each other in the same study and give an overview of the significant results. Finally, we give a summary of the findings of significant results regarding the comparison of all studies.

### 4.1. Statistical and Visualization Methods

In order to compare the results of the studies with each other, we started with an analysis of each study individually. For this purpose, we computed ANOVA tests of each factor. If this resulted into findings with *p* < 0.01, *p* < 0.05 and *p* < 0.10 (whereas we consider the latter as a mere indication), we will give the F-statistic *F* it's corresponding *p*-value, as well as the effect-size ω^2^. Afterwards, we compared each condition to each other condition with an ANOVA Test using the Bonferroni correction (Abdi and Salkind, 2007). We used the Shapiro-Wilk test (Shapiro and Wilk, 1965) in order to test the null hypothesis that the data was drawn from a normal distribution.

In order to visualize the data, we use box plots from the seaborn library (Waskom et al., 2017). The box extends from the Q1 to Q3 quartile values of the data, with a line at the median (Q2). The whiskers extend from the edges of a box to show the range of the data. We use the same colors and naming system as introduced in Figure 1.

### 4.2. Factor Overview

First of all, we want to provide an overview to the collected data. We decided to visualize the factors time, errors, QUESI (usability), NASA-TLX (task load) in a comparison of all sample data in boxplots, as SART and ISONORM were only recorded in the last two experiments. Furthermore, only the third experiment conducted a grounding analysis. For additional information to the third experiment see Aschenbrenner et al. (2018b). This section only contains the ANOVA comparison of the conditions within each study.

#### 4.2.1. Task Duration

**Figure 9:**
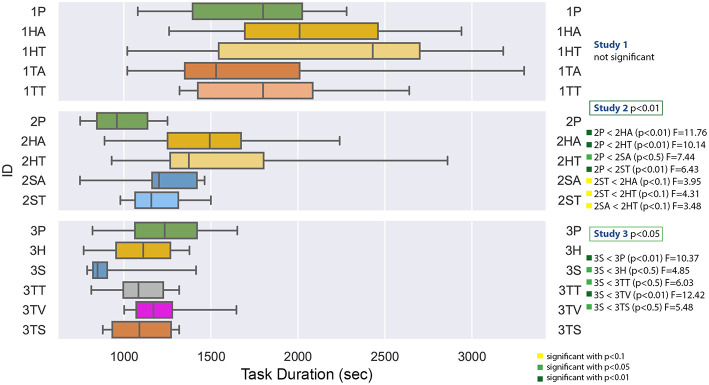
A boxplot of the repair duration in seconds comparing all experiments.

Figure 9 displays the task duration data for all studies. The ANOVA tests compare the conditions of each study and the significant result are summarized on the left. The measured task duration for the first study had a very high variance, so that there were no significant results. This is probably a result of the fact, that the student participants clearly lacked domain knowledge—some of them had problems to use the screwdriver in this setting and needed a very long time, the longest experiment took 55 min (for 1TA, tablet PC with audio). The shortest task duration was 17 min for 1HT (head-mounted display with text). This is in contrary to the median, where 1HT has the highest and 1TA the lowest value. The result of the Shapiro-Wilk test is not statistically significant, which indicates that the residuals are normally distributed.

The second study was conducted by the technician apprentices and showed a significant difference of the task duration with *F*_(4, 45)_ = 4.687 with *p* = 0.0030 and ω^2^ = 0.2278. Planned *post-hoc* testing, using the Bonferroni correction, revealed that the condition 2P (paper instructions) has a significantly lower task duration than all of the other conditions. Furthermore, the spatial AR methods, especially the spatial AR with text (2ST) seem to induce a shorter duration than the other conditions. The Shapiro-Wilk test is significant with *p* = 0.0064.

The task duration comparison of the third study in the collaborative setting with technician apprentices was significant *F*_(5, 54)_ = 3.316 with *p* = 0.0110, ω^2^ = 0.1618. The *post-hoc* testing showed a significantly lower task duration for the projection based method 3S. The Shapiro-Wilk test was not significant.

#### 4.2.2. Errors

**Figure 10:**
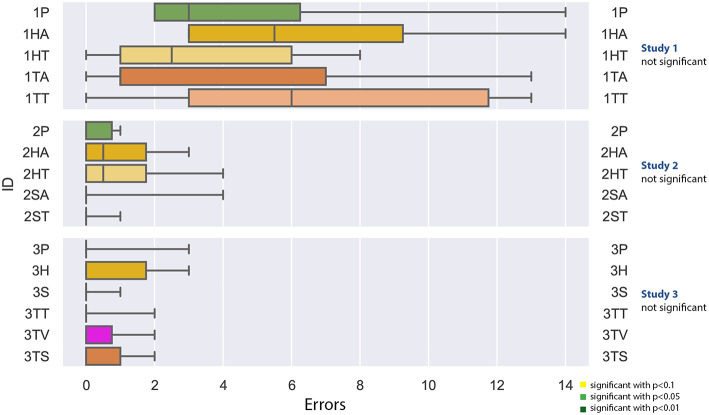
A boxplot of the recorded errors, comparing all experiments.

As noted in Figure 10, none of the studies yielded significant results. For the first experiment, the amount of errors deviated between 0 and 14, whereas the highest median of error was generated with the tablet PC with text (1TT). In the second study, the highest amount of error was four, in the third studie, the highest amount of errors was 3. For all studies, the Shapiro-Wilk test was not significant.

#### 4.2.3. Usability

**Figure 11:**
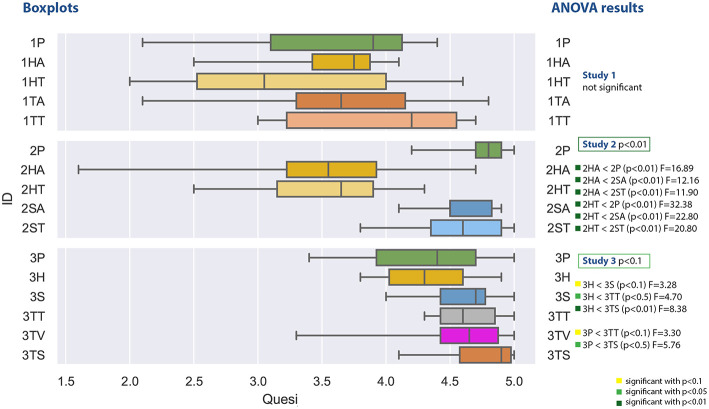
A boxplot comparing all experiments regarding the values from the QUESI questionnaire measuring usability.

The values in Figure 11 represent the mean of the different QUESI factors as specified in the questionnaire definition (Anja and Jörn, 2010). The higher the values, the better the overall usability has been rated.

The first study does again not yield any significant results. Participants reported values which led to an calculated overall value between 2 and 4.8. The lowest mean can be found at the head-mounted display with text support (1HT) and the highest for the tablet PC with Text (1TT). The Shapiro-Wilk test is not significant.

The second study generated QUESI values between 1.6 and 5. The ANOVA comparison was significant with *F*_(4, 45)_ = 12.237 with *p* = 8.1^−07^ and ω^2^ = 0.473. The *post-hoc* test revealed a significant lower usability for the head mounted display for both audio and text support compoared to the other conditions. The Shapiro-Wilk test is significant with *p* = 0.0048.

The third study comparison of the usability questionnaire was significant with *F*_(5, 54)_ = 2.181 with *p* = 0.0697 and ω^2^ = 0.0895. The *post-hoc* comparison showed significant low values for the head-mounted display for both text and audio in comparison to the spatial augmented reality and the tablet PC applications. The tablet PC application with screenshot achieved the best rating with respect to the median comparison (not significant). Values within the third study varies between 3.3 and 5. The Shapiro-Wilk test was significant with *p* = 0.0247.

#### 4.2.4. Task Load

**Figure 12:**
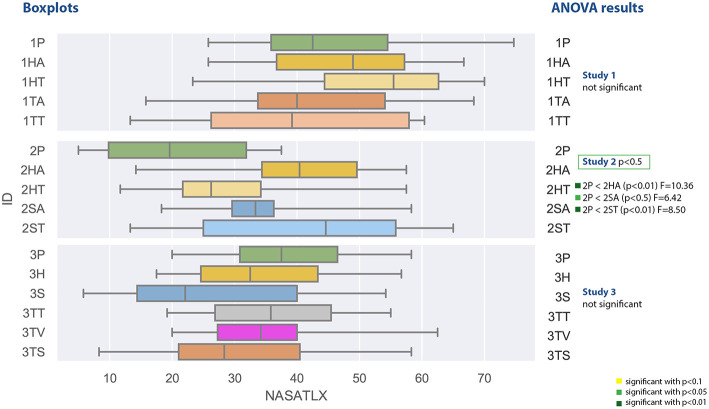
A boxplot comparing all experiments regarding the values from the NASA-TLX questionnaire measuring task load.

The task load values measured with the NASA TLX are shown in Figure 12 and spread between 13.3 and 74.7 for the first experiment. They are computed as a mean of different subjective ratings and a higher value means a high task load. The first study did not yield any significant results and the Shapiro-Wilk test was not significant as well. The highest median was reported for the head-mounted display with text instructions, the lowest median have been measured for the tablet PC with text instruction.

The second study measured task load values between 5 and 65 and was significant with *F*_(4, 45)_ = 3.338, *p* = 0.0178 and ω^2^ = 0.158. The *post-hoc* test revealed significant lower values for the paper based instruction (2P) in comparison to the head mounted device with audio (2HA) and both spatial awareness applications with text (2ST) and audio (2SA). The Shapiro-Wilk test was not significant.

The results of the third study with respect to the task load are not significant. The contain values between 5.8 and 62.5 and the lowest median was achieved by the spatial augmented reality application. The Shapiro-Wilk test was not significant.

### 4.3. Summary of the Individual Studies

#### 4.3.1. First Study: Students Single-Mode

The study was performed with the help of the proband system of the institute for human-computer-media Würzburg. Fifty students of the field of study human computer systems (18) and media communication (32) have been recruited, consisting of 27 females and 23 males with an average age of 21 years. No participant has ever performed a similar repair task before or had been accustomed to the experiment workflow.

Although none of the factor comparisons was significant, the application using head-mounted display (especially the one with text instructions) had a higher task duration than the paper instructions, whereas the task completion time with the application with a tablet PC was shorter. With respect to the error values, both HMD with audio and tablet with text resulted in more error than the paper instructions, whereas the HMD with text and the tablet with audio instructions had lower values. The use of the tablet-based version with text instructions was rated slightly better regarding usability and task load and in the qualitative results, although we could not find a significant confirmation. Notably, the participants had very different mechanics foreknowledge.

#### 4.3.2. Second Study: Apprentices Single-Mode

The study was performed with the help of the Franz-Oberthür-Schule in Würzburg. Fifty technical apprentices have been recruited, 4 of which were female. All participants were acquainted to similar repair tasks and perform them on a regular basis. They have not been familiar with AR before.

The study showed a significant superiority of the paper instructions compared to the AR applications, the participants needed less time in all comparisons. Additionally, the paper instructions provided significant less task load compared to all other applications except the HMD with text representation. The total amount of errors is very low and does not show any significant differences.

Comparing the different two AR implementations, the SAR applications performed better than the HMD applications. There are indications (with *p* < 0.10), that the SAR applications need less time. With respect to usability, both variants of the head-mounted display have been accessed with significant lower usability.

Also the qualitative feedback showed that the apprentices are used to work with paper instructions and the task is relatively easy for them. They did need too much guidance and experienced the Augmented Reality applications as less efficient.

#### 4.3.3. Third Study: Apprentices Collaboration-Mode

The user study was carried out with *N* = 50 participants (48 male, 2 female) which were between 19 and 26 years old. The average age was 21.1 years (*SD* = 1.9 years). All participants were recruited from the technician school Franz-Oberthür-Schule in Würzburg. Ninety-two percent of the participants said that they need to solve mechanic tasks on a daily basis and spend approximately 23 h per week with those tasks. Sixty-four percent said that they solve those tasks from time to time in teamwork and 34% said that they work in a team regularly. The individual results of this study have already been published in Aschenbrenner et al. (2018b).

Regarding task duration, the projection-based SAR (3S) was significantly better than all other conditions. With regards to usability, the head-mounted display application significantly underperformed the SAR condition as well as tablet with tracking and with screenshots. Also the phone condition significantly underperformed the latter two tablet conditions. Neither the analysis of the errors nor of the task load was significant.

Summarizing the results of Aschenbrenner et al. (2018b) the analysis of grounding analysis showed that all visual media performed significantly better than the phone condition.

### 4.4. Comparison of All Studies

A valid question is, whether the three studies can be compared at all. Study 1 clearly has a completely different user group, and whereas study 2 used a single-user setting, study 3 was conducted within a collaborative setting. Thus, any results from the following analysis must take into account, that these facts will eventually superimpose any findings of differences between devices and implementations. Because of this reason, the authors refrained from conducting a device-specific comparison (for example all HMD condition in comparison to all tablet PC conditions).

We calculated a *post-hoc* comparison with Bonferroni correction, if the ANOVA test of the comparison of the factor over all three studies was significant. For each factor, we provide a table with the comparison results, where the significance level is highlighted with colors (*p* < 0.10 (yellow), *p* < 0.05 (light green) and *p* < 0.01 (dark green); ns is white and stands for “not significant”).

**Figure 13:**
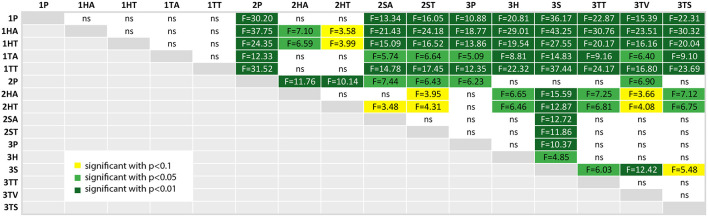
A table comparing the measured task duration of all conditions. The ANOVA results was significant with *p* < 0.01 and *F*_(15, 144)_ = 9.843 and ω^2^ = 0.4532. The F-statistics of the *post-hoc* test are depicted for *p* < 0.10 (yellow), *p* < 0.05 (light green) and *p* < 0.01 (dark green); ns, not significant. The Shapiro-Wilk test is significant with *p* = 0.0004.

For example, a comparison of the factor task duration between the paper instructions of the second experiment and the phone condition of the third experiment can be found in the row 2P and the column 3P in Figure 13. If the corresponding box-plots from section 4.2 are considered additionally to the *post-hoc* test tables (in this case the Figure 9), the relation between the two conditions can be immediately seen: The values for 2P are lower than 3P (and we know from the table, that this is significant with *p* < 0.05 and *F* = 6.43).

#### 4.4.1. Task Duration

As expected from Figures 9,13 confirms, that the first experiment (first 5 rows) took significantly more time for the tasks than the other two experiments, except for the head-mounted display conditions in the second experiment—although the student participants using the same HMD applications (1HA and 1HT) still underperformed compared to the apprentices (2HA and 2HT). As seen in Figure 9, the variance of the task duration is very high for the first experiment. The reason for both findings is probably the lack of domain knowledge of the student participants which led to the decision to repeat the experiment with domain experts.

The baseline condition for the first and second experiment is the paper instruction (1P and 2P), and in case of the third experiment the phone condition (3P). The significant results of the second experiment are visible in the overall-comparison: the paper condition 2P outperforms all of the other second experiment conditions. Furthermore, 2P task duration is significantly shorter than all of the first experiment, including the paper instructions. Finally, this condition 2P took significantly less time than the baseline of the third experiment (3P), which means that the task takes longer with phone support (without shared visual context) than with paper instructions, which could be expected due to grounding effort.

The head-mounted display with text used by the apprentices took significant longer than all of the third experiment conditions (except the phone condition 3P). The SAR application (3S) had a significant shorter task duration than all of the other conditions except 2P.

#### 4.4.2. Errors

Errors have been specified by domain experts. As it can be seen in the boxplot in Figure 10, the student participants made more errors than the domain experts. Although both experiments with apprentices show a very low error rate, the head-mounted display led to more errors than the other conditions. In the collaborative setting, some errors are omitted anyways, as the expert will correct the working person, if he or she is committing a visual error, for example plugging cables in the wrong intake. Still, proper mounting (so that no cable or plug is loose) cannot be controlled remotely.

**Figure 14:**
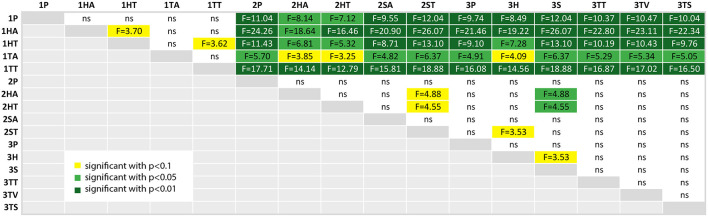
A table comparing the measured error amount of all conditions. The ANOVA results was significant with *p* < 0.01 and *F*_(15, 144)_ = 8.920 and ω^2^ = 0.4261. The F-statistics of the *post-hoc* test are depicted for *p* < 0.10 (yellow), *p* < 0.05 (light green) and *p* < 0.01 (dark green); ns, not significant. The Shapiro-Wilk test is not significant.

As already mentioned above, the student participants in the first study made a lot of errors. This is confirmed in Figure 14, where all conditions of the study 1 resulted in significantly more errors.

The other significant difference was found between the tablet PC conditions of study 2 (2HA and 2HT) in comparison to the spatial AR in the third study (3S). The latter resulted in significantly less errors.

#### 4.4.3. Usability

**Figure 15:**
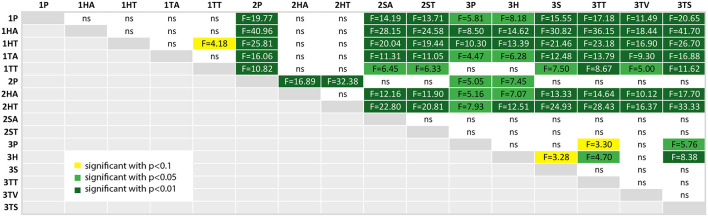
A table comparing the subjective QUESI questionnaire results for all conditions. The ANOVA results was significant with *p* < 0.01 and *F*_(15, 144)_ = 8.799 and ω^2^ = 0.4223. The F-statistics of the *post-hoc* test are depicted for *p* < 0.10 (yellow), *p* < 0.05 (light green) and *p* < 0.01 (dark green); ns, not significant. The Shapiro-Wilk test is significant with *p* = 0.0039.

At first, already Figure 11 showed a clear difference between both user groups, which is now confirmed in Figure 15. The students in the first experiment had a larger variance in this subjective measure, which can also be due to the lack of domain knowledge. Furthermore, the values in study 1 are clearly lower than the other two experiments (except for the HMD conditions in study 2).

It is interesting, that the perceived usability of the head mounted display in the second study (2HA and 2HT) was rated significantly lower than all of the other conditions in study 2 and also in study 3. If we regard the plots at Figure 11, the QUESI values of the HMD conditions for the first and second experiment is comparable, although both user groups are different.

The head mounted-display in the third study (3H) performed significantly better than the HMD conditions in the first and second study, but was rated with a lower usability than the three other AR conditions of the third experiment: 3S (*p* < 0.10), 3TT and 3TS (3TV only provides video without augmentation).

The highest values were achieved by the paper instruction condition in the second study (2P) and the tablet screenshot (3TS) condition in the collaborative setting. The control condition of the second experiment (2P) was rated with a higher usability than the phone condition (3P) and the collaborative HMD condition (3P). The screenshot variant of the third experiment (3TS) yields significantly higher (ergo better) values than phone condition (3P) and the HMD condition (3H).

#### 4.4.4. Task Load

**Figure 16:**
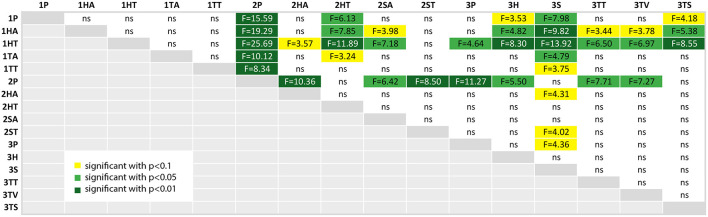
A table comparing the measured subjective NASA-TLX questionnaire results of all conditions. The ANOVA results was significant with *p* < 0.01 and *F*_(15, 144)_ = 2.994 and ω^2^ = 0.1575. The F-statistics of the *post-hoc* test are depicted for *p* < 0.10 (yellow), *p* < 0.05 (light green) and *p* < 0.01 (dark green); ns, not significant. The Shapiro-Wilk test is significant with *p* = 0.0095.

Figure 12 compares the measured values of the NASA-TLX questionnaire, which measures task load. Again we can clearly see that the first experiment participants perceived a higher task load than the other participants, but in general the range of the task load is widespread. The results from the *post-hoc* comparison of the task load values depicted in Figure 16 show still some interesting results, although a lot of comparisons did not yield significant results.

At first, the task load of the paper instructions in the second experiment (2P) is significantly lower than the results of the first experiments. It also is significantly lower than the other conditions of experiment 2 except tablet with text (2TT). Additionally, it still outperforms all of the conditions of the third experiment but the screenshot condition (3TS) and the SAR condition (3S).

The highest values, and thus the highest perceived task load, were achieved by the head-mounted display condition with text in the first experiment (1HT). This is not significant for the first study, but for all but the HMD with audio in the second study (2HA, here *p* < 0.01) and the second study SAR with text condition.

There is an indication that the SAR condition (3S) has less task load than the SAR condition with text in experiment 2 (2ST) and the control condition of experiment 3 (3P), as both have *p* < 0.1. The task load of 3S is significantly lower than all the conditions in study 1 (except 1TT where *p* < 0.1) and there is an indication *p* < 0.1, that it is also lower than audio based HMD implementation in experiment 2 (2HA).

### 4.5. Comparison of the Tasks

**Figure 17:**
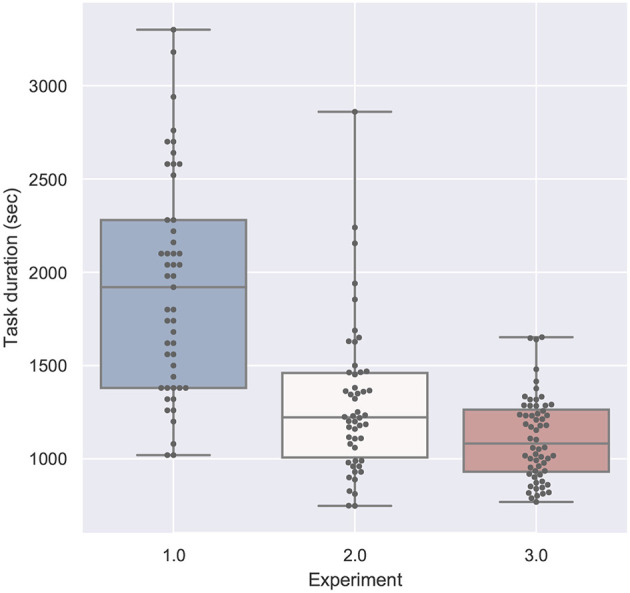
Task duration compared by experiment is significant with *p* < 0.01 with *F*_(1, 158)_ = 94.166 and ω^2^ = 0.368. Shapiro-Wilk test is significant.

**Figure 18:**
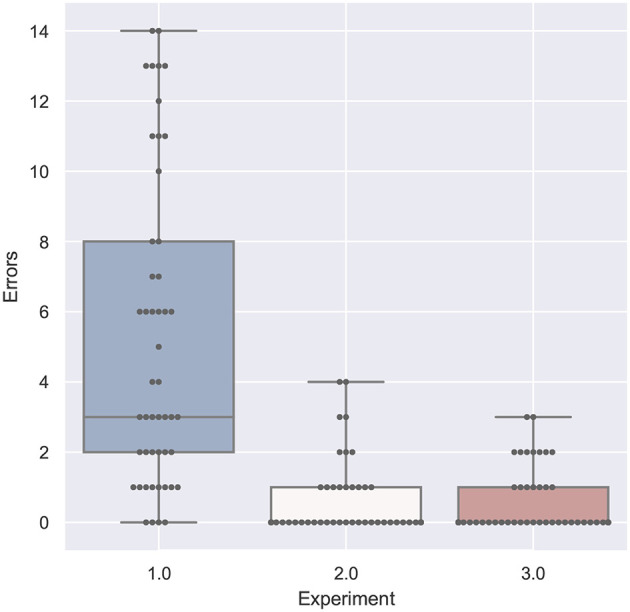
Errors compared by experiment is significant with *p* < 0.01 with *F*_(1, 158)_ = 77.169 and ω^2^ = 0.323. Shapiro-Wilk test is significant.

**Figure 19:**
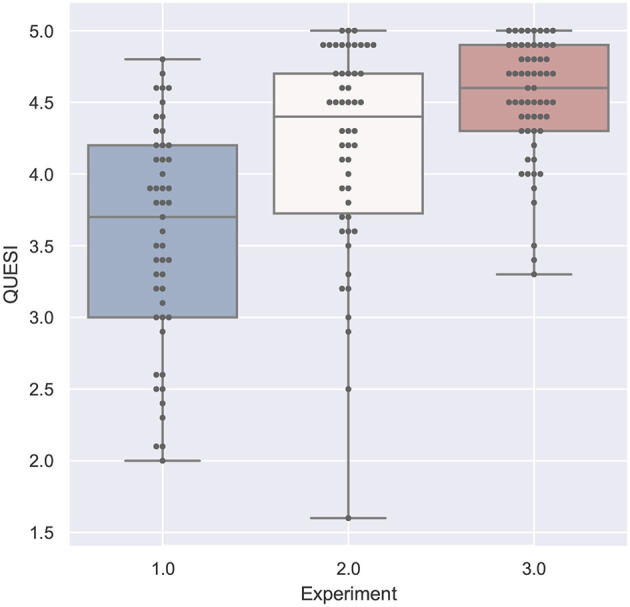
QUESI compared by experiment is significant with *p* < 0.01 with *F*_(1, 158)_ = 57.779 and ω^2^ = 0.262. Shapiro-Wilk test is significant.

**Figure 20:**
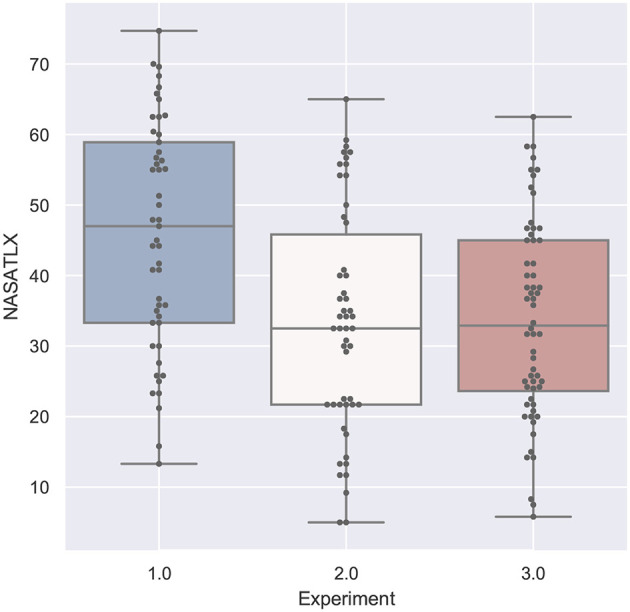
NASA-TLX compared by experiment is significant with *p* < 0.01 with *F*_(1, 158)_ = 15.247 and ω^2^ = 0.0818. Shapiro-Wilk test is not significant.

Figures 17–**20** show a comparison of the four main factors between all experiments. Furthermore, for each comparison, an ANOVA test has been computed, which was significant in all cases. F-statistics are displayed next to the Figures.

Comparing both single-mode studies (study 1 and 2), the different training background is visible: the students need much more time than the technicians (see Figure 17), caused more errors (see Figure 18) and experienced a higher task load (see Figure 20) and a lower usability (see Figure 19).

If we compare the two studies performed by participants with domain knowledge (study 2 and 3), the analysis of the error in Figure 18 and the task load in Figure 20 are very similar. With respect to task duration, the mean of the second study (single-mode) is higher than the collaborative case (study 3) as can be derived from Figure 17. The median QUESI value for the collaborative case in Figure 19 is higher than the single user case, but both differences are not significant.

## 5. Conclusion

Coming back to our questions from the beginning: Which device should be used, with which visualization and interaction methods? Admittedly, it is still hard, to deduct general guidelines from the results presented in this paper.

The first user study was carried out with student participants and did not result in any significant result. If a decision would need to be withdrawn from this, very wage tendency toward the tablet PC applications and against the used HMD could be detected from the comparison of the mean values from task duration and task load. Also the qualitative results from the interviews confirmed, that the student experienced the task as “too hard” and apparently the applications were not able to compensate this in a sufficient or measurable amount.

The lack of domain knowledge which led to the results of the first study was compensated in the second study with a different user group (technician apprentices). Here a very clear result showed, that the paper instructions performed significantly best in all factors. This is maybe a result of the fact, that the apprentices are very used to such tasks. The task was apparently “too easy,” so that no assisting technologies were needed. In a comparison of the two used AR implementations HMD (2HA and 2HT), we find a significant lower usability rating than for the other applications and some indications with *p* < 0.01, that the projection-based applications (2SA and 2ST) also lead to a lower task duration. In order to support this group, an instruction application, which only supports if necessary, would be best, but the entire approach of “guiding the user through the process” as implemented here does not seem promising.

In a comparison of the first and second study, the different user group show a strong effect in all factors but task load. All conditions of the second study except the HMD conditions (2HA and 2HT) perform better than the conditions of the first study. This shows the weakness of the used HMD and furthermore, that a student participant group is not suited for these kind of experiments.

In the third study, a collaborative setting was used with technician apprentice participants. The baseline was phone support, but this led to the longest task duration and the highest task load (not significant). The video-based tablet application, which did provide shared visual context but did not allow to share visual cues did perform only slightly better. The projection-based condition (3S) performed significantly better than all of the other conditions of this study regarding task duration. The task load comparison was not significant, but the mean of the 3S condition was lower than the other conditions. With respect to usability, the head mounted display had lower values than the Augmented Reality implementations on the tablet PC (3TT and 3TS). The results of the grounding analysis presented in Aschenbrenner et al. (2018b) showed, that significant differences in how the worker and the supervisor communicate and interact with each other can be found, if the visual media are compared with phone support. From the results of the third study, one would point a potential industrial user toward projection-based SAR or (being conservative) to the usage of a tablet PC with screenshot and visual cues.

A comparison of the second and third study showed the differences between single-mode and collaborative usage. If we compare the baseline conditions, the paper instruction baseline of the second study (2P) required a significantly shorter time, a lower task load and a higher perceived usability. Whereas additional support for the user was apparently not beneficial in the single-mode, the collaborative mode showed the necessity for using additional Augmented Reality applications. Especially the spatial AR application (3S) was even significantly faster than all of the applications of the second study including the paper instructions. This shows the benefit of collaboration, when provided with a suitable medium.

Our conclusion of this comparison: AR support can be very important and helpful for a collaborative setting. Though we were not able to show a clear superiority of AR support within the single mode repair setting, the shared visual context was crucial for the collaborative setting. A remote human helper via phone alone is not superior to paper or automated instructions. But as soon as a shared visual context is added within a collaborative Mixed Reality (CMR) system, we are able to show superiority in both task and human factor metrics.

Some findings are only visible, if presented in such a comprehensible overview:

First of all, most of the user studies in the literature are carried out with students. In this publication, we can clearly see that this does not provide enough value, even if (and with smaller group size this is more probable) it yields significant results. The differences between both user groups are only visible, if presented in comparison of the same task.

Secondly, the three studies have been carried out on the same task, which allows at least some comparability with respect to the performance of different AR devices as noted above.

Finally, the main finding is, that AR can provide additional value especially for collaborative cases compared to single usage. This can be due to the fact that humans can adapt much more on the visual indications that are needed for a special working step and for a specific context. This highlights on the one hand the importance of Collaborative Mixed Reality and on the other hand the need for adaptive context-sensitive communication in single-user modes. This also can only be deducted from a comparison of all three studies.

## 6. Discussion

The starting point for the user studies presented in this paper was the question for the right device for supporting maintenance and repair operations in an active production line. This demand emerged 2012 by our industry partners in the research project “MainTelRob” and provided us with a real repair application case and the corresponding hardware, the switch cabinet of an industrial robot. Based on the results above, we can identify the following recommendations for future work:

1. **Importance of real applications:** Although we do not doubt that the use of domain neutral topics like LEGO blocks can be very helpful for determining underlying psychological principles, we strongly recommend research on real world applications in order to assist industry to choose the best option in the current AR/VR hype.
2. **Importance of variety testing:** We want to encourage other researchers to take the time to implement different AR methods for a specific application and to benchmark different frameworks on the same real world application. In our eyes, only proofing the superiority of the own new solution to a baseline will soon not be enough to drive AR research forward.
3. **Importance of end users:** Most user studies use students. Similar to Dey et al. (2018) we want to encourage more end user research, as we can clearly see the different results on the studies at hand. Untrained people struggle with the use of tools, leading to a high variance within the measured performance. The task is “too demanding" for this group to be able to show clear results. Trained professionals have already developed strong preferences and are very used to similar tasks. The clear result for a significantly better performance of the paper instructions shows that the task is eventually “too easy” for them. We limited our study to one specific age range, so it would be certainly interesting to have some results from older participants.
4. **Need for benchmarking tasks:** The user studies presented in this paper have been gathered over a couple of years, starting in 2014. This is clearly one reason for the poor performance of the used head-mounted display, because the device simply has not been mature enough. We did not want to change it during this study, in order to be able to compare the results over different studies. Nevertheless, it would be most interesting to benchmark newer devices with the same task. As we cannot easily transport the used switch cabinet to other researchers, we propose to introduce some kind of benchmarking tasks which can be performed in each larger user study and allow a comparability between different devices and also between different studies.
5. **Need for CMR research:** During the third user study in the collaborative setting we used highly adaptive instructions provided by another human being instead of a computer. With this we could clearly show a better performance compared to the control scenarios of paper instructions and telephone scenario. This strongly supports future research into collaborative mixed-reality (CMR). It especially shows that AR in a collaborative setting already makes sense, although the technical maturity of single-mode systems might still lack the requirements of real conditions outside the lab. This is also shown by applications like Vuforia Chalk, Skype+Hololens, Hololens settings, which start to acquire a large user base.

## Ethics Statement

We do not have considered an ethics committee, this has been discussed with the editor and included in the text.

## Author Contributions

ML advised during the conceptual design. FL contributed with his SAR application. AÇ assisted on the framing of the comparison. JV and ML assisted with user study design. KS and ML enabled project and working context. KS enables project and working context. SL contributed with his knowledge on collaborative AR with regards to user studies, argumentation and data analysis.

### Conflict of Interest Statement

The authors declare that the research was conducted in the absence of any commercial or financial relationships that could be construed as a potential conflict of interest.
